# Exploring the Mediating Role of Self-Regulation in Bullying Victimization and Depressive Symptoms among Adolescents: A Cross-Regional and Gender Analysis

**DOI:** 10.3390/healthcare12151486

**Published:** 2024-07-26

**Authors:** Qi-Lu Huang, Wing-Shan Ho, Ho-Nam Cheung

**Affiliations:** 1 Department of Social and Behavioural Sciences, City University of Hong Kong, Hong Kong, China; wsho76-c@my.cityu.edu.hk; 2 Department of Social Work and Social Administration, The University of Hong Kong, Hong Kong, China

**Keywords:** bullying victimization, depression, self-regulation, adolescents, cross-cultural study, gender differences

## Abstract

This study explores the mediating role of self-regulation in the relationship between bullying victimization and depressive symptoms among adolescents, considering the moderating effects of gender and region. A cross-sectional analysis was conducted with 3984 adolescents aged 12–18 from the United Kingdom, Hong Kong, Taiwan, and the Netherlands. Data were collected via an online survey administered through Qualtrics. The survey included validated measures such as the Illinois Bullying Scale (IBS) to measure bullying victimization, the Adolescent Self-Regulatory Inventory (ASRI) to measure self-regulation, and the Patient Health Questionnaire (PHQ) to measure depression. The SPSS macro PROCESS was employed for data analysis, with model 4 used for testing the mediating effects of self-regulation and model 1 for assessing the moderating effects of gender and region. The results demonstrated significant associations between bullying victimization, self-regulation, and depressive symptoms. Self-regulation mediated the positive association between bullying victimization and depression, with notable variations across genders and regions. Specifically, male students in Hong Kong exhibited an increased susceptibility to depression when subjected to bullying. These findings underscore the protective role of self-regulation in mitigating the adverse effects of bullying victimization on adolescent mental health. Implications for interventions and prevention strategies targeting adolescent depression are discussed.

## 1. Introduction

Adolescent depression is a critical public health issue with significant long-term consequences, including impaired social functioning, academic difficulties, and an increased risk of substance abuse and suicidal behavior [1]. Among the various factors contributing to adolescent depression, bullying victimization has emerged as a potent risk factor. Bullying, defined as repeated aggressive behavior intended to harm an individual perceived as vulnerable, can lead to severe psychological distress and long-lasting emotional scars [2]. This study explores the mediating role of self-regulation in the relationship between bullying victimization and depressive symptoms among adolescents while also examining the moderating effects of gender and region.

The prevalence of adolescent depression and bullying victimization varies globally, influenced by cultural, social, and environmental factors. According to the World Health Organization (WHO) [3], approximately 1.1% of adolescents aged 10–14 and 2.8% of those aged 15–19 years old experience depression worldwide. A meta-analysis of 41 studies found a 2.6% prevalence of depressive disorder among children and adolescents [4]. Another review indicated that major depressive disorder had an 8% prevalence rate, with elevated depressive symptoms increasing from 24% (2001–2010) to 37% (2011–2020) [5]. Similarly, bullying victimization is also widespread, with approximately 36% of adolescents experiencing bullying [6]. A study covering 48 countries reported a high prevalence of past-12-month suicide attempts (10.7%) and past-30-day bullying victimization (30.4%) [7]. These statistics underscore the global significance of depression and bullying as public health issues among adolescents and highlight the need for effective interventions tailored to different cultural contexts.

The negative impact of bullying on mental health has been extensively documented, with numerous studies highlighting the association between bullying and depression in adolescents [8,9]. One prominent theory is the stress process model, which posits that stressors such as bullying lead to adverse mental health outcomes through the erosion of personal and social resources [10]. According to this model, bullying may serve as a chronic stressor that can deplete an adolescent’s emotional and psychological resources, resulting in depressive symptoms [11,12]. Moreover, when bullying takes place in school, it creates an unsafe and hostile atmosphere that poses a threat to the mental development, interpersonal relationships, and self-esteem of adolescents. According to the interpersonal risk model, exposure to unfriendly social environments can trigger negative emotions such as depression [13]. A comprehensive review conducted by Dennehy et al. [14] highlighted the need for further research to explore the factors that contribute to the relationship between bullying victimization and depression. This research provides valuable insights into potential interventions and prevention strategies.

Self-regulation, defined as the ability to control one’s thoughts, emotions, and behaviors in pursuit of long-term goals, is a crucial factor in managing stress from bullying and preventing depression [15]. Brown [16] defined self-regulation as the ability to plan, monitor, and direct one’s behavior in changing situations. Self-regulation is also conceived as a self-correcting procedure when faced with discrepancies or indications of imminent danger [17]. Recent research has indicated that promoting self-regulation can serve as a protective factor against depression [18] and bullying [19], leading to fewer depressive symptoms and better academic performance. However, the combined effect of these factors remains to be investigated.

Self-regulation may act as a buffer against the negative impact of bullying on depression, and this potential role can be understood through several theoretical frameworks. Bandura’s social cognitive theory of self-regulatory systems [20] proposes that self-regulation not only mediates the effects of external influences but also provides the basis for purposeful action in directing change. According to this theory, individuals with strong self-regulation skills are better equipped to cope with the negative impacts of bullying, thereby reducing the likelihood of developing depression. Additionally, regulatory focus theory, developed by Higgins [21,22], further elaborates on the mechanisms of self-regulatory activities by distinguishing between two types of goal pursuit: promotion focus, which involves striving for positive outcomes, and prevention focus, which involves avoiding negative outcomes. Adolescents who can effectively employ both regulatory focuses are more likely to navigate the challenges posed by bullying, thereby mitigating its adverse effects on mental health.

The prevalence and impact of bullying can vary across different regions and among different genders, highlighting the need for a closer examination of cross-regional and gender variations in adolescent mental health and bullying experiences [23]. Understanding these variations is crucial for developing effective prevention and specific intervention strategies. Cross-region variations have been well documented; cultural, societal, and environmental factors play a role in shaping the prevalence and nature of bullying as well as its impact on adolescent mental and physical health [24,25]. Factors such as social acceptability, cultural conceptualization of bullying, educational systems, social support networks, and implementations of national policies and programs contribute to the prevalence of bullying and its effects on mental health outcomes among adolescents in different regions [24,26].

Regarding gender variations, research consistently shows that boys and girls may experience bullying differently in terms of bullying experiences and mental health outcomes, with girls more likely to face relational aggression, while boys often experience more direct forms of bullying [2]. A cross-national study in 40 countries found that boys were more likely than girls to be involved in both bullying others and being bullied [26]. These gender differences may also extend to the mental health consequences of bullying, with some studies suggesting that boys may be more susceptible to developing depressive symptoms and suicidal ideation in response to bullying victimization [27].

However, there is still a need for further investigation to fully understand the cross-regional and gender variations in adolescent mental health and bullying experiences. Regarding the potential moderator role of regions, there is a lack of understanding about how the cultural context, educational systems, and geopolitical context of different regions could affect the association between bullying victimization and depression. In terms of the possible moderator role of gender, there is a gap in understanding regarding the impact of gender role expectations and social norms on the moderating effect. The findings from this study aim to provide valuable insights for policymakers, educators, and mental health professionals to develop targeted interventions and prevention strategies that address the specific needs and challenges faced by adolescents in different regions and across genders.

**Figure 1:**
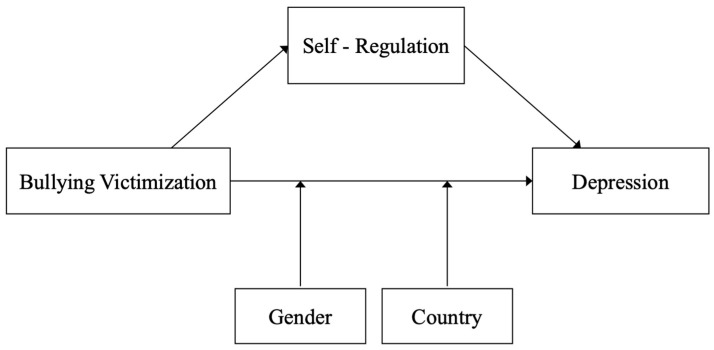
The conceptual framework of the current study.

The present study aimed to investigate the relationship between bullying victimization and depression among adolescents from diverse regions. Additionally, we aimed to explore the mediating role of self-regulation in this relationship. Furthermore, we sought to examine the moderating effects of gender and region on the association between bullying victimization and depression. The present study’s hypotheses were as follows and are visualized in Figure 1 through a proposed conceptual framework.

Based on the existing literature, the following hypotheses were formulated:

1. Correlation Hypothesis: Bullying victimization has a positive correlation with depression. It was hypothesized that an adverse life event that potentially poses a threat to the mental development of adolescents and contributes to the development of depression;
2. Mediation Hypothesis: Self-regulation mediates the association between bullying victimization and depressive symptoms among adolescents. It was hypothesized that self-regulation would serve as a mechanism through which the experience of bullying victimization influences the development of depressive symptoms such that lowering levels of self-regulation would amplify the impact of bullying victimization on depressive symptoms;
3. Moderation Hypothesis: Gender and region moderate the relationship between bullying victimization and depressive symptoms. Specifically, it was hypothesized that the association between bullying victimization and depressive symptoms would vary depending on gender and region. For instance, males may exhibit a stronger association between bullying victimization and depressive symptoms compared to females, and the strength of this association may differ across regions.

By examining these hypotheses, we aim to enhance our understanding of the mechanisms underlying the relationship between bullying victimization and depression among adolescents, with implications for the development of targeted interventions and support programs for secondary school students facing bullying victimization.

## 2. Materials and Methods

### 2.1. Participants and Procedures

A total of 3984 participants between the ages of 12 and 18 were recruited from four regions: the United Kingdom (UK), the Netherlands (NL), Hong Kong (HK), and Taiwan (TW), using the Qualtrics online platform (see Table 1). This study is part of an international collaboration focused on examining adolescent behavior and mental health within diverse cultural contexts. Due to practical considerations and the feasibility of accessing a diverse sample of adolescents, we employed convenience sampling to recruit participants. The recruitment process for the study took place in 2022 and involved multiple channels, such as advertising on school websites, social media outreach, and utilizing the research team’s professional networks. The four study areas were chosen to encompass a range of cultural contexts, educational systems, and geopolitical contexts. As a result, the number of participants varied across regions based on the availability and willingness of schools and adolescents to engage in the research.

The sample included 1055 secondary school students from the United Kingdom (26.5%), 959 from the Netherlands (24.1%), 925 from Hong Kong (23.2%), and 1045 from Taiwan (26.2%). Individuals who were illiterate in written English and traditional Chinese were excluded from the study. In the United Kingdom, the sample consisted of 1055 secondary students with a mean age of 16.56 (*SD* = 1.49), and 60.6% of them were females. The majority of participants from the United Kingdom were Caucasian (65.6%). Among them, 21.1% reported having a history of mental disorders in the past two weeks. In the Netherlands, there were 959 secondary students with a mean age of 16.07 (*SD* = 1.57), and 67.9% of them were males. The majority of participants from the Netherlands were Caucasian (84.3%). Among them, 5.6% reported a history of mental disorders in the last two weeks. In Hong Kong, the sample consisted of 925 participants with a mean age of 15.87 (*SD* = 1.8), and 65% were male. The majority of respondents from Hong Kong identified themselves as Asian (88%). Only 2.5% of participants from Hong Kong reported experiencing mental disorders in the last two weeks. In Taiwan, there were 1045 participants with a mean age of 16.22 (*SD* = 1.56), and 55.6% were male. The majority of Taiwanese participants identified as Asian (92.3%). Among them, 2.7% reported a history of mental illness in the past two weeks.

Ethical approval for the study was obtained from the university’s ethical committee, and informed consent was obtained from schools, parents, and students before data collection commenced. Participants provided informed consent by checking the appropriate box on the online survey platform. Confidentiality and anonymity of participants’ responses were maintained throughout the study.

### 2.2. Measures

Bullying Victimization. Bullying victimization was assessed using the Illinois Bullying Scale (IBS), a validated instrument developed by Espelage and Holt [28]. The IBS comprises 18 items designed to measure experiences of bullying victimization and perpetration. The scale includes three subscales: bullying perpetration (example item: *“I helped harass other students”*), bullying victimization (example item: *“I got hit or pushed by other students”*), and fighting (example item: *“I got into a physical fight”*). Each item is rated on a scale ranging from 0 (never) to 4 (seven or more times), with higher scores indicating a greater frequency of the respective experiences. For the purpose of this study, only the bullying victimization subscale of the IBS was utilized. The Cronbach’s alpha coefficient for the bullying victimization subscale in our study was found to be 0.89, indicating high internal consistency.

Self-regulation. Self-regulation was assessed using the Adolescent Self-Regulatory Inventory (ASRI) developed by Moilanen [29]. This inventory consists of 36 items designed to evaluate teenagers’ ability to regulate their emotions, behaviors, and interests across short-term and long-term domains. Short-term self-regulation focuses on immediate goals and involves aspects such as attention to details and emotional management. In contrast, long-term self-regulation pertains to managing impulses, interests, and emotions toward future targets, such as career aspirations and financial planning. Participants rated the accuracy of each statement on a Likert-type scale ranging from 1 (not completely true for me) to 5 (very true for me). The Cronbach’s alpha coefficient for the ASRI in our study was 0.94.

Depression. Depressive symptoms were assessed using the Patient Health Questionnaire (PHQ), specifically the PHQ-9, a self-administered diagnostic tool for depression [30]. The PHQ-9 comprises nine items corresponding to the DSM-IV criteria for depression, with responses scored on a scale ranging from 0 (not at all) to 3 (nearly every day). Total scores, obtained by summing the item scores, indicate the severity of depressive symptoms, with higher scores reflecting greater symptom severity. A cutoff score of ≥10 has been shown to have 88% sensitivity and specificity for major depression in the general medical population [30]. In our study, the PHQ demonstrated high internal consistency, with a Cronbach’s alpha coefficient of 0.91.

Demographic Information. Demographic data, including gender, age, ethnicity, region of residence, history of mental disorders, and history of mental disorder treatments, were collected via an online survey administered to participants.

### 2.3. Data Analysis

The data analysis utilized the SPSS macro PROCESS by Hayes [31]. Initially, we examined the mediating effects of self-regulation on the association between bullying victimization and depression using model 4 of the SPSS macro PROCESS for simple mediation analysis via linear regression. Covariates, including age (12–18 years), gender (ordinal: 1 = male, 2 = female, 3 = other gender), region (ordinal: 4 = United Kingdom, 5 = The Netherlands, 6 = Hong Kong, 7 = Taiwan), and history of mental disorders (dummy: 1 = no), were controlled for in the prediction of self-regulation and depression. Bootstrapping procedures (5000 samples) were applied to evaluate the significance of the indirect effect, generating a 95% bias-corrected bootstrap confidence interval (CI).

Subsequently, the moderating effects of gender and region on the association between bullying victimization and depression were tested using model 1 of the SPSS macro PROCESS. Simple slope analysis [32] was employed to identify significant moderating effects. This method allowed for the investigation of the relationship between two variables in the presence of a third variable while controlling for covariates.

## 3. Results

### 3.1. Correlational Analyses

Table 2 reports descriptive statistics and zero-order intercorrelations for major study variables and related variables, including bullying victimization, depression, self-regulation, age, gender, region, and history of mental disorders. Self-regulation was negatively related to bullying victimization (*r* = −0.13, *p* < 0.01) and negatively correlated to depression (*r* = −0.22, *p* < 0.01). Depression had a positive relationship with bullying victimization (*r* = 0.53, *p* < 0.01).

### 3.2. Mediation Analyses

The study assessed the mediating role of self-regulation in the relationship between bullying victimization and depressive symptoms. The result revealed a significant indirect effect of the impact of bullying victimization on depression (*b* = 0.032, *t* = 6.47, 95% CI [0.023, 0.042]). Furthermore, the direct effect of bullying victimization on depression in the presence of self-regulation as the mediator was also found to be significant (*b* = 0.658, *p* < 0.001). Hence, self-regulation partially mediated the relationship between bullying victimization and depression. The mediation analysis summary is presented in Table 3.

### 3.3. Cross-Region and Gender Comparisons: Moderation Analyses

We first assessed the moderating role of gender on the relationship between bullying victimization and depression severity (PHQ). The test of unconditional interaction shows the change in R-Sq due to interaction (x × w) is significant (*p* < 0.001). The result revealed that females have a higher PHQ than males. Differences in PHQ between females and males were significant (*b* = 0.939, *t* = 5.504, *p* < 0.001). The result also revealed that those who consider themselves to be of other genders have a higher PHQ than males. Differences in PHQ between other genders and males were insignificant (*b* = 1.220, *t* = 1.065, *p* > 0.05). Both interaction effects are significant. The impact of bullying victimization on bullying severity in females and other genders is considerably different (lower) from that of males.

**Figure 2:**
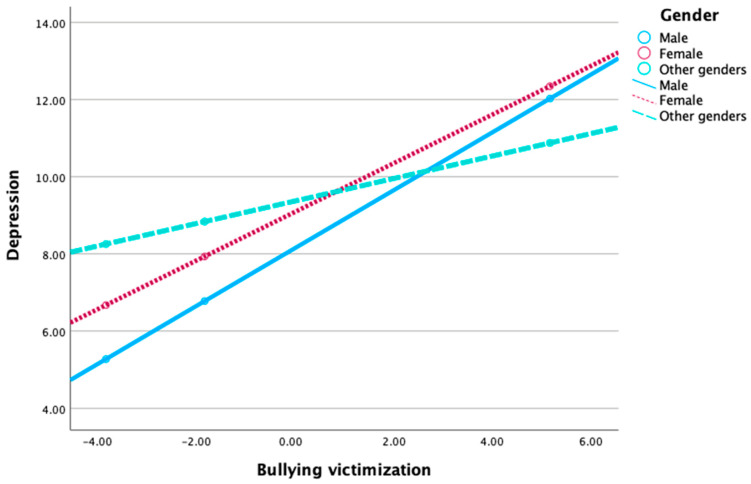
The interaction effect between gender and bullying victimization on depression severity.

The results of the simple slope analysis conducted to better understand the nature of the moderating effects are shown in Figure 2. As shown in Figure 2, the line representing males is much steeper. This indicates that the impact of bullying victimization on depression severity is much stronger in males compared to females and those who consider themselves to be of other genders.

The study assessed the moderating role of the region on the relationship between bullying victimization and depression severity (PHQ). The test of unconditional interaction shows that the change in R-Sq due to interaction (x × w) is significant (*p* < 0.001). The results revealed that the NL has a lower depression severity in comparison to the UK (*b* = −0.933, *t* = −3.884, *p* < 0.001). Also, HK has a lower depression severity in comparison to the UK, and the differences in PHQ between HK and the UK were significant (*b* = −1.559, *t* = −6.379, *p* < 0.001). Furthermore, TW has a significantly lower PHQ than the UK (*b* = −1.825, *t* = −7.553, *p* < 0.001). Three interaction effects were significant (*p* < 0.001), and the impact of bullying victimization on depression severity in the NL, HK, and TW is significantly different (higher than) from the UK.

**Figure 3:**
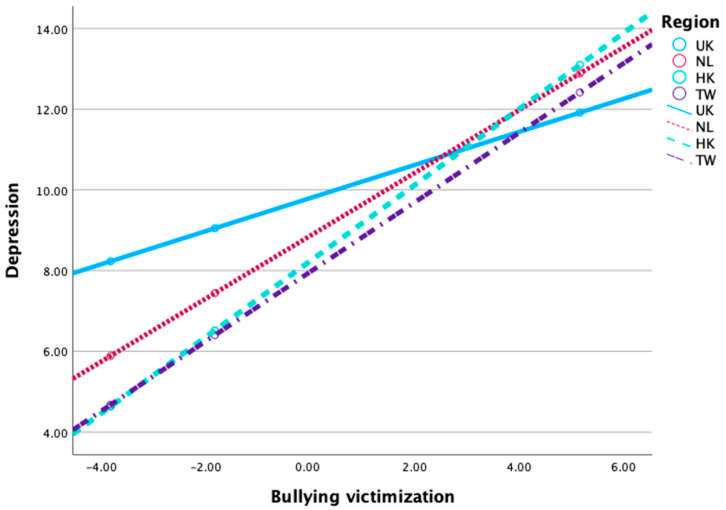
The interaction effect between region and bullying victimization on depression severity.

The results of the simple slope analysis conducted to better understand the nature of the moderating effects are shown in Figure 3. As shown in Figure 3, the line representing Hong Kong is slightly steeper. This indicates that the impact of bullying victimization on depression severity is slightly greater in Hong Kong compared to other regions.

## 4. Discussion

The present study found a significant positive association between bullying victimization and depressive symptoms among adolescents, indicating that higher levels of bullying are predictive of more severe depressive symptoms. Particularly, in this relationship, self-regulation emerged as a significant mediator, suggesting that adolescents with better self-regulation skills are less likely to experience severe depressive symptoms despite being bullied. Furthermore, the study identified gender and regional differences, with the impact of bullying victimization on depression being more pronounced in Hong Kong males and adolescents. These findings support both the mediation and moderation hypotheses, underscoring the complex interplay between individual and contextual factors in adolescent mental health.

Our findings align with the existing literature that underscores the significant relationship between bullying victimization and depression in adolescents [8,9]. A meta-analysis corroborated that bullied adolescents are significantly 2.77 times more likely to suffer from depression than those who are not bullied [33]. This study extends these findings by demonstrating the mediating role of self-regulation, supporting the notion that self-regulation can buffer against depression by enhancing adolescents’ coping mechanisms [15,20].

The study highlights the pivotal role of self-regulation in mitigating the adverse effects of bullying on depression. Adolescents who can effectively manage their thoughts and emotions are better equipped to cope with the stress of being bullied [18]. The findings align with the regulatory focus theory [21,22], which elucidates that self-regulation involves both promotion and prevention strategies, which help individuals navigate stress and achieve their goals. In this context, self-regulation enables adolescents to shift their focus towards positive outcomes and away from the negative impacts of bullying, thereby reducing depressive symptoms [34,35,36]. Bandura’s social cognitive theory [20] further supports this by highlighting the role of self-regulatory systems in mediating external influences and guiding purposeful actions. Adolescents with robust self-regulatory skills are better equipped to cope with bullying, as they can effectively manage their emotional responses and maintain psychological well-being. These findings emphasize the need for incorporating self-regulation training into anti-bullying interventions, empowering adolescents to handle bullying experiences more resiliently to prevent depression. Integrating these theoretical insights into practical interventions can significantly enhance the mental health outcomes for bullying victims, fostering a supportive environment for adolescent development.

Our cross-regional and gender analyses further enrich the literature, confirming cultural and societal influences on bullying experiences and mental health outcomes [24,26]. The study revealed significant regional differences, with bullying’s effects on depression being more severe in Hong Kong. This could be due to the limited mental health support systems and interventions for adolescents in Hong Kong compared to other regions. Despite a “zero tolerance policy” towards school bullying introduced by the Education Bureau, Hong Kong reports high bullying rates (the worst among 54 counterparts) and insufficient mandatory reporting, which may contribute to the underreporting and inadequate handling of bullying cases [37,38]. These findings suggest a pressing need to bolster mental health support services and effective anti-bullying interventions in Hong Kong, especially for bullying victims.

Additionally, gender-specific differences in bullying experiences and responses are consistent with previous research, which shows boys are more likely to suffer severe psychological impacts from bullying [27,39]. This may relate to societal norms and expectations that discourage boys from expressing vulnerability and seeking help, thus exacerbating the mental health impacts of bullying [40,41]. The dominance of masculine norms that emphasize toughness and self-reliance likely complicates emotional regulation and increases susceptibility to depression among bullied boys [42]. These results highlight the necessity of gender-sensitive interventions that address the unique needs of male bullying victims, particularly in regions like Hong Kong with limited resources. Additionally, it emphasizes the significance of promoting healthy coping strategies and challenging societal gender norms to mitigate the negative impact of bullying victimization on mental health.

This study holds significant implications for refining school-based interventions, particularly for victims of bullying. Traditional whole-school approaches often overlook individualized psychological support, neglecting the unique needs of victims and perpetrators [43]. Strategies aimed at reducing self-blame and empowering victims to speak out against victimization may alleviate depressive symptoms and diminish engagement in bullying behaviors. For example, cognitive–behavioral interventions, specifically targeting victims of school bullying, have demonstrated efficacy in promoting active coping and reducing depressive–anxious symptoms [44]. Our findings underscore the potential efficacy of integrating therapeutic strategies focused on cultivating self-regulation to mitigate maladaptive self-focus and depressive symptoms associated with bullying victimization. Gender-specific considerations, especially in regions like Hong Kong with higher male depression rates, call for collaborative efforts between school administrators and mental health professionals to foster a supportive learning environment for all students.

In a nutshell, this study contributes significantly to understanding adolescent depression by elucidating the mediating role of self-regulation and examining regional and gender variations. The large, diverse sample enhances the generalizability of these findings, underscoring the universal importance of addressing bullying and its mental health consequences across different cultural contexts. The key findings reveal that self-regulation plays a crucial role in mitigating the effects of bullying on depression, emphasizing the need for interventions that enhance self-regulatory skills. Regional and gender differences highlight the importance of culturally and gender-sensitive approaches in addressing bullying and its mental health impacts. Future research should explore longitudinal designs to establish causal relationships and investigate qualitative aspects to understand cultural disparities better. Overall, integrating self-regulation training into anti-bullying programs and addressing gender-specific needs can significantly improve mental health outcomes for adolescents.

While this study offers valuable insights into the mediating role of self-regulation in the context of bullying victimization and depressive symptoms among adolescents, several limitations warrant consideration and opportunities for future research. Firstly, the cross-sectional design precludes causal inference, necessitating longitudinal or cross-lagged panel designs to elucidate temporal relationships. Secondly, the comparative analysis across regions underscores the need for qualitative exploration to deepen our understanding of cultural disparities. Future research could refine the conceptualization of bullying victimization by distinguishing between aggressive and pure victims’ profiles, such as their mental resources and distinct experiences of psychological distress, and facilitating targeted intervention strategies [45]. Such efforts promise to enhance the efficacy of anti-bullying initiatives and psychological interventions for bullying victimization. Additionally, the current study found that self-regulation only partially mediates the relationship between bullying victimization and depression, and the effect is not particularly strong. Therefore, it would be beneficial for future research to explore other potential mediators that could serve as coping mechanisms. One potential mediator could be critical thinking. By developing critical thinking skills, adolescents can better assess and respond to adverse situations, potentially mediating the relationship between victimization and depressive symptoms. Likewise, considering there are a wide range of adverse health and psychosocial problems related to bullying victimization, future research could address other mental health outcomes such as anxiety, low self-esteem, post-traumatic stress disorder (PTSD), and suicidal ideation [46,47].

## 5. Conclusions

In summary, our study adds to the expanding body of literature on adolescent mental health by highlighting the mediating role of self-regulation in the complex interplay between bullying victimization and depressive symptoms. By examining data from diverse cultural contexts, we gained insights into the universal nature of these phenomena while also acknowledging the nuanced variations shaped by regional and gender differences. Our findings underscore the importance of adopting a comprehensive approach to addressing adolescent depression that considers both individual and contextual factors. Interventions aimed at enhancing self-regulatory skills and creating supportive environments may offer effective strategies for mitigating the detrimental effects of bullying on adolescent mental health. Nevertheless, further research is warranted to explore the long-term implications of these findings and to develop evidence-based interventions tailored to the specific needs of diverse teenage populations. By continuing to investigate these dynamics, we can advance our understanding of adolescent mental health and inform targeted interventions that promote resilience and well-being among vulnerable youth.

## Figures and Tables

**Table 1:** Demographic table by region.

Variables	United Kingdom	The Netherlands	Hong Kong	Taiwan
	(*n* = 1055)	(*n* = 959)	(*n* = 925)	(*n* = 1045)
	26.5 (%)	24.1 (%)	23.2 (%)	26.2 (%)
Age				
Mean	16.56	16.07	15.87	16.22
*SD*	1.49	1.57	1.8	1.56
Gender				
Female	639 (60.6)	301 (31.4)	324 (35.0)	464 (44.4)
Male	394 (37.3)	651 (67.9)	601 (65.0)	581 (55.6)
Ethnicity				
Asian	147 (13.9)	71 (7.4)	814 (88.0)	965 (92.3)
Caucasian	692 (65.6)	808 (84.3)	88 (9.5)	67 (6.4)
Black	114 (10.8)	35 (3.6)	13 (1.4)	3 (0.3)
Hispanic	4 (0.4)	10 (1.0)	3 (0.3)	2 (0.2)
Mixed Race	16 (1.5)	27 (2.8)	7 (0.8)	8 (0.8)
History of mental disorders				
Yes	223 (21.1)	54 (5.6)	23 (2.5)	28 (2.7)
No	832 (78.9)	905 (94.4)	902 (97.5)	1017 (97.3)
Mental disorder treatments				
Yes	92 (8.7)	141 (14.7)	8 (0.9)	26 (2.5)
No	963 (91.3)	818 (85.3)	917 (99.1)	1019 (97.5)

**Table 2:** Descriptive statistics and intercorrelations of key variables.

Variables	*M*	*SD*	1	2	3	4	5	6	7
1. Bullying victimization	3.83	4.11	-						
2. Depression	8.58	6.4	0.53 **						
3. Self-regulation	109.88	10.92	−0.13 **	−0.22 **					
4. Age	16.19	1.63	−0.06 **	0.05 **	−0.01 **				
5. Gender	1.45	0.51	−0.09 **	0.09 **	−0.02 **	0.14 **			
6. Region	5.49	1.14	−0.22 **	−0.28 **	0.1 **	−0.09 **	−0.13 **		
7. History of mental disorder	1.08	0.28	0.12 **	0.32 **	−0.14 **	0.11 **	0.19 **	−0.25 **	-

Note: *N* = 3984; ** *p* < 0.01. *M* and *SD* represent the mean and standard deviation, respectively.

**Table 3:** Mediation analysis summary.

Relationship	Total Effect	Direct Effect	Indirect Effect	Bootstrap 95% Confidence Interval		t-Statistic	Conclusion
				Lower Bound	Upper Bound		
Bullying victimization -> Self-regulation -> Depression	0.690	0.658	0.032	0.023	0.042	6.47	Partial

## Data Availability

The data that support the findings of this study are available from the primary corresponding author, H.-N.C., upon reasonable request.
